# PERVALE-S: a new cognitive task to assess deaf people’s ability to perceive basic and social emotions

**DOI:** 10.3389/fpsyg.2015.01148

**Published:** 2015-08-07

**Authors:** José M. Mestre, Cristina Larrán, Joaquín Herrero, Rocío Guil, Gabriel G. de la Torre

**Affiliations:** ^1^Laboratorio de Inteligencia Emocional, Departamento de Psicología, Universidad de CádizCadiz, Spain; ^2^Centro de Educación Especial para Sordos, Junta de AndalucíaJerez de la Frontera, Spain

**Keywords:** emotional perception ability, deaf, assessing emotional perception, emotional knowledge in deaf people, emotional perception measure, adaptation criteria in deaf people

## Abstract

A poorly understood aspect of deaf people (DP) is how their emotional information is processed. Verbal ability is key to improve emotional knowledge in people. Nevertheless, DP are unable to distinguish intonation, intensity, and the rhythm of language due to lack of hearing. Some DP have acquired both lip-reading abilities and sign language, but others have developed only sign language. PERVALE-S was developed to assess the ability of DP to perceive both social and basic emotions. PERVALE-S presents different sets of visual images of a real deaf person expressing both basic and social emotions, according to the normative standard of emotional expressions in Spanish Sign Language. Emotional expression stimuli were presented at two different levels of intensity (1: low; and 2: high) because DP do not distinguish an object in the same way as hearing people (HP) do. Then, participants had to click on the more suitable emotional expression. PERVALE-S contains video instructions (given by a sign language interpreter) to improve DP’s understanding about how to use the software. DP had to watch the videos before answering the items. To test PERVALE-S, a sample of 56 individuals was recruited (18 signers, 8 lip-readers, and 30 HP). Participants also performed a personality test (High School Personality Questionnaire adapted) and a fluid intelligence (*Gf*) measure (RAPM). Moreover, all deaf participants were rated by four teachers for the deaf. Results: there were no significant differences between deaf and HP in performance in PERVALE-S. Confusion matrices revealed that embarrassment, envy, and jealousy were worse perceived. Age was just related to social-emotional tasks (but not in basic emotional tasks). Emotional perception ability was related mainly to warmth and consciousness, but negatively related to tension. Meanwhile, *Gf* was related to only social-emotional tasks. There were no gender differences.

## Introduction

Perceiving emotions is an important ability to build emotional intelligence (EI). As it was many times stated, the first branch (of 1997 Mayer and Salovey EI model) is clearly defined as perceiving emotions accurately in oneself and others (Mayer et al., 2004). Perception is a cognitive process, which has traditionally been divided into two interdependent directions: top–down and bottom–up (Kosslyn and Smith, 2006). According to Galotti (2008), data-driven or bottom–up processing occurs when an interpretation emerges from the data. Perceiving emotional expressions “accurately" must thus be largely data-driven because it should reflect precision in the interpersonal relationships where emotions have an important role (Roberts et al., 2006; MacCann and Roberts, 2008; Grunes et al., 2013). In the case of bottom–up processing of emotional stimuli, the interpretation of an emotional expression scene needs to be determined mostly by information from the senses rather than expectations. Nevertheless, in many situations, knowledge or expectations are involved in emotional perception. This process is named schema-driven or top–down processing. Top–down perception processes encompass the mental abilities to drive both the observation and external stimuli into a priori concepts of an understanding exploration (Goldstein, 2008). As Bruner (1973) summarized, people perceive “beyond the information given” constantly in our mental processes, such as learning to add assumptions and supplemental information derived from past experience to the evidence of our senses to understand the emotional world. Likewise, the accurate perception of emotions should encompass both top–down and bottom–up processes.

To understand how deaf people (DP) perceive emotions, it is necessary to develop an instrument that considers how DP process information (input) from the emotional stimuli. While hearing people (HP) perceive emotions through different channels (iconic and echoic sense organs), DP use mostly iconic emotional inputs. Moreover, there are differences in the perception of iconic-emotion inputs between DP and HP. Strong evidence of this difference comes from the activation of the neural circuits for recognizing emotions. Signers (DP) have to identify other factors in facial expressions (other than the emotion) to compensate for the hearing impairment. Indeed, HP activate the right superior temporal sulcus (STS) while DP show bilateral STS activation, during emotional perception tasks (McCullough et al., 2005). This instrument should distinguish both basic and social emotional expressions according to Spanish codes for DP, as we explain in the Section “Materials and Methods.”

The distinction between basic and social emotions have been based in the remarkably consistent findings provided by two experienced research teams led by Ekman and Izard about facial expressions and the distinction between basic and social emotions (see Ekman, 2006, for a deeper explanation). According to Ekman (1992), basic emotions have nine characteristics, which distinguishes from the rest of emotions. These well-known characteristics are: distinctive universal signal (1), physiology (2), and universals in antecedents events (3); also have presence in primates (4), coherence among emotional response (5), a quick start (6), a short duration (7), automatic appraisal (8), and, finally, unexpected occurrence (9). However, social emotions are defined as affective states that depend on the social context and arise when people interact each other, and they are related to the self as well (Lamm and Singer, 2010).

As emotion perception in the brain differs between DP and HP, the cognitive process involved (top–down or bottom–up) could differ as well. For example, Rieffe et al. (2003) indicated that deaf children understand emotional emergence differently from their hearing peers. The authors proposed that hearing children are more interested on why an emotion arose, while deaf children seemed more attentive to the achievement of a desired emotional state, without reasoning much on why that state occurred (Rieffe et al., 2003). Another study investigated how DP process to a particular story. The authors found that DP kept less details than hearing peers, and that DP tended to make interpretations different from those of HP (Cambra et al., 2010). Meanwhile, Dyck and Denver (2003) and Dyck et al. (2004) have found differences between DP and HP in emotional iconic information processing. DP showed deficits in every single scale in comparison with HP, even when compared in the same age intervals (Dyck and Denver, 2003; Dyck et al., 2004). Thus, iconic perception is critical in the understanding of how DP perceive their environments (Ziv et al., 2013). Moreover, social emotion development involved more cognitive effort and time than basic emotions (Arsenio, 2003).

Evidence of how DP and HP differ in the iconic perception of emotions comes from a study of Letourneau and Mitchell (2011). The authors tested 12 HP and 12 DP who were beginners in American Sign Language to examine whether the specialized experience can alter typically observed gaze patterns. Participants had to “judge the emotion and identity of expressive faces (including whole faces, and isolated top and bottom halves), while accuracy and fixations were recorded” (Letourneau and Mitchell, 2011, p. 563). All individuals recognized faces more accurately from top compared to bottom halves, and emotional expressions from bottom compared to top halves. HP paid more attention to the bottom half when they had to evaluate an emotion. In contrast, DP fixated equally on the top and bottom halves regardless of task demands (identity or emotion). The authors suggested that DP could maximize their ability to gather information from expressive faces.

Another important deficit in emotional perception ability (EPA) in DP is prosody or intonation (Most et al., 1999). Important meaningful emotional information is conveyed in prosody, but DP are unable to access it. However, what happens when DP learn to speak? One way could be through cochlear implants. Wiefferink et al. (2013) compared performance in emotion recognition tasks between children with cochlear implants and hearing children. They found that (a) children with cochlear implants were less competent in the emotion recognition tasks than hearing peers, and (b) despite having cochlear implants, these children had some aspects of emotion recognition tasks affected, including their abilities to understand emotions conveyed non-verbally. A possible explanation is that children with cochlear implants may not have effectively developed prosody. However, it was evident that these children were able to understand language, albeit with “metallic” sounds (Kermit, 2009).

Another way for DP to learn to speak is through learning to read lips and spoken language. A few years ago, several European countries (including Spain) promoted various laws for developing language abilities in DP, especially bilingualism (sign language and oral language). An ability to read lips and develop oral language could be learned simultaneously or successively (Acosta-Rodríguez, 2003). Being able to lip-read could be more advantageous in comparison to only signing. Ludlow et al. (2010) showed that only signing deaf children had problems in identifying emotions, and that this inability affected their social skills negatively. Both the delay in language acquisition and/or absence of oral language lead DP to lack opportunities for talking about their personal experiences, and therefore, fewer chances to develop normal emotional perception. Indeed, earlier acquisition of language in DP (signing and/or lip-reading) improved their scores in Theory of Mind (ToM) tasks (a top–down perception) on interpreting what emotions were experienced by others (Meristo et al., 2007; Glickman, 2009; Morgan et al., 2014). However, other studies did not find differences between DP and HP regarding their emotional, social, and communicative development (Masataka, 1996; Peterson and Slaughter, 2006; Meronen and Ahonen, 2008).

The relationship between EPA and social adaptation has been described many times (see Kumschick et al., 2014, for instance). Hosie et al. (2000) developed a study to examine how hearing and deaf children understand socially displayed rules, and how they express and conceal emotions. Regarding displaying rule knowledge, the authors did not find differences between deaf children and hearing peers. However, deaf children reported having difficulties concealing happiness and anger. The difficulty in concealing these two emotions might be disadvantageous to deaf children in some social situations, where both emotions need to be regulated properly. An interesting study about how deaf and hearing children’s parents rated their children’s academic and social aspects found that hearing children and their parents rated the children’s friendships more positively than did deaf children and their parents. However, the authors found that deaf children and deaf parents rated the children’s social skills more positively. The authors suggested that as these children used sign language at home, the social skills learned at home were generalized to the school context (Marschark et al., 2012). Similar findings have been observed in other cultures, such as Argentina (Ipiña et al., 2010) or Brazil (Prietch and Filgueiras, 2013).

The next point to discuss is the theoretical framework to study emotion perception in DP and how to measure it. Many emotional perception measures were developed and validated based on emotional knowledge (EK); especially if this tool is for use on children under social adaptation criteria (Denham, 1986; Morris et al., 2013; Mestre et al., 2014). EK is a theoretical construct which includes mainly: both recognizing and labeling expressions of emotions and understanding their transition from their causes to their consequences (Morgan et al., 2009). However, some revised studies on the perception of emotions in DP (especially with children) used ToM tasks as criteria (as autism studies do), rather than social adaptation criteria. Dyck and Denver (2003) pointed out that children with language impairment have difficulties, among others, in: (a) second-order ToM tasks (understanding false beliefs; do not appear until children are older, at around 3–5 years of age; see Hughes and Leekam, 2004 for a review); (b) recognizing non-verbal emotional expressions; and (c) matching facial expressions (especially with emotional intonation, for hearing impairment).

In contrast, EK includes a set of factors interrelated to emotional perception, such as age, gender, and verbal intelligence, due to the understanding that at certain ages cognitive and emotional development are mutually dependent (Morris et al., 2013). This is precisely the problem in assessing verbal intelligence. Building a tool to assess EPA (or EK) in DP should encompass the entire construct of EK. It is thus necessary to pay attention to the special characteristics discussed above. Most existing instruments assume that DP could understand written language. However, there is in fact a high illiteracy rate among the deaf population (Massone and Baez, 2009). Could a fluid intelligence (*Gf*) test be used instead of a verbal one? Using a *Gf* test would also allow us to examine the role of *Gf* on emotional perception. Another question is the appropriate age at which to administer the tool; for testing our developed tool, we used a sample ranging in age from 12 to 30 years. This is because we are interested in identifying standard performance, instead of how deaf children will score it.

Another factor often ignored in emotional perception among DP is personality. In principle, DP should not have personality traits different from HP. However, HP tend to misunderstand this reality, and tend to assign some traits as stubborn or distrustful to the deaf (Rodríguez-Ortiz, 2005). According with this idea, some studies have pointed out that emotional perception (measured as a part of the MSCEIT, Mayer et al., 2004) had a low-to-moderate relationship with some personality traits, especially Neuroticism (negatively), Openness and Consciousness (both positively; see Mayer et al., 1990; Day and Carroll, 2004; Lopes et al., 2012). However, some studies showed that personality traits and mood states are involved in the emotional perception processing (for instance, Rusting, 1998). Some personality traits stimulate certain mood states that then influence on the emotional perception. As example, Knyazev et al. (2008) found that personality could systematically influence in how people perceive facial expressions of other people. For instance, they pointed out that some traits, both agreeableness and conscientiousness, prepossessed to perceive the faces in a friendly way. However, anxiety and aggressiveness overrate or misunderstand the intentions of other people.

Finally, an emotional perception instrument for DP should consider how emotional stimuli are to be presented. Regarding emotional facial expressions, Fernandez-Dols (2013) strongly recommended to “restore a balance between the top–down and bottom–up strategies” for displaying emotional expression stimuli. An emotional expression “is a continuous flow of muscular movements from bodies moving in a three-dimensional world which produces events with flexible and context-dependent meanings” (Fernandez-Dols, 2013, p. 6). Following this advice (and from others Elfenbein, 2013; Hassin et al., 2013), we included videos of emotional expression. The videos used as emotional stimuli were of a lip-reading DP.

In order to address the issues explained above, we designed a pilot study to assess DP’s EPA using a software created for DP. Following the suggestions from Leon et al. (2011) about how to proceed in pilot studies, we prefer to address objectives rather than research questions and hypothesis. There are several purposes of conducting a pilot study: one of them is assessment procedures. Another relevant question, a “pilot study does not provide a meaningful effect size estimate for planning subsequent studies due to the imprecision inherent in data from small samples” (Leon et al., 2011, p. 626).

The objectives of the pilot study were:

(a) To analyze the emotional perception achievement among groups (signers, lip-reading deaf, and hearing), which involves making a confusion matrix^1^ of both basic and social emotions, according to group performance;
(b) To check whether the instrument fits the EK construct (should be related to age and intelligence; gender would be not involved because of sample age); and
(c) Based on EPA and personality studies, we expect to find:
  (c.1) Regarding personality: positive relationships of EPA with verbal intelligence and some personality traits (consciousness and warmth). However, it is expected negative relationships between EPA and the next traits: tension, excitability, dominance, apprehension, and self-sufficiency.
  (c.2) Regarding adaptation criteria: positive relationships between EPA and all adaptation criteria, excepting unrest, which is expected a negative relationship.

## Materials and Methods

### Participants

A total of 56 individuals were identified according to the language used (signers, lip-reading DP, and HP). Participants were required to have normal or corrected-to-normal vision, and no learning disabilities (including illiteracy). We decided not to include children with cochlear implants because most of them were under 12 years old. All parents of participants under 18 years old signed a consent form for their children’s inclusion in the pilot study. Deaf participants (*n* = 26) were included in the research under the supervision of the director of the *Centro de Educación Especial para Sordos* (Special Education Center for Deaf), Jerez de la Frontera (Southern Spain). Hearing participants were collected from the same geographical area, and had similar characteristics to the deaf sample (age range: 12–30 years, 60% male). We decided not to include two deaf participants because their parents were also deaf. Participants received a brief report about their outcomes. **Table 1** summarizes the demographic characteristic of the participants.

**Table 1:** Frequency, Sex, and Age information by group.

Groups	*N*	Sex frequency (female/male)	AGE		
			*M*	*SD*	Range
Signers	18	7/11	21.06	6.31	12–32
Lip-readers	8	2/6	16.63	5.34	12–29
Hearing people (HP)	30	11/19	23.4	4.48	12–32
Total	56	20/36	21.68	5.65	12–32

*Signers are deaf people (DP) without lip-reading abilities who learnt Spanish Sign Language (SSL); Lip-readers are DP who learnt both to speak and read lips, also in SSL; HP have no trouble hearing*.

The ethics committee of the deaf school (November 25, 2013) approved the pilot study after we presented the project to them. All participants and parents of under-18 children signed both an agreement of collaboration and a consent to participate in this research (December 30, 2013).

### Measures

#### Emotional Perception

PERVALE-S (*Test de Tareas Cognitivas de Percepción y Valoración de Emociones en sordos*, Test of cognitive tasks for perceiving and valuing emotions in deaf; Herrero et al., 2009).

PERVALE-S is an improved version of a software specially designed to assess both basic and social EPA in DP. The instrument developed both basic and social emotional expressions. Although there is no unanimity, we considered as basic emotions: fear, joy, sadness, disgust, anger, and surprise (especially last one is quite controversial). For our emotional perception instrument, we decided to include anxiety, jealousy, envy, and embarrassment as social emotions. However, it is necessary to explain briefly how jealousy and envy are different (we did not consider to include guilty, so embarrassment should not be confused with guilty), and both emotions have different expressions too in Spanish signal code for DP. According to Salovey and Rodin (1988), jealousy and envy could have quantitative differences besides other semantic considerations. However, other authors have demonstrate that both emotions have qualitative differences. Parrott and Smith (1993) introduced new methodologies in order to clarify how both emotions reveal differences. In their experiments, they pointed out that envy was “characterized by feelings of inferiority, longing, resentment, and disapproval of the emotion.” However, jealousy “was characterized by fear of loss, distrust, anxiety, and anger” (Parrott and Smith, 1993, p. 906). The social-emotional expression items of the instrument show different stimuli regarding the Spanish-deaf expression for both emotions.

The answer scale in the first version of the instrument had to be changed from five levels to three (1: a little; 2: a lot; and 3: without emotion), because DP had problems differentiating beyond three levels of intensity of an emotional expression stimulus. In the previous study, we also discovered that DP had difficulties discriminating between close frequency adverbs (e.g., the difference in meaning between “very” and “quite a bit” was not apparent to the deaf sample). Another important difference between PERVALE-S and a regular EK tool is the stimuli presented. To more accurately identify the emotion expressed, DP need to watch the upper body (especially arm movements) rather than just the emotional expression on the face. Thus, the emotional stimuli presented should include “simultaneous or successive facial movements linked to affective reactions”—involving mostly bottom-up perception processes—and “appraisals, social motives, or strategies of regulation, but also to cognitive processes or cultural conventions”—involving especially top–down perception processes (Fernandez-Dols and Crivelli, 2013, p. 27). Nevertheless, showing just the face might not be enough for DP—emotional expression stimuli for the deaf must show moving arms and faces, all in a videotaped emotional expression (Herrero et al., 2009).

**FIGURE 1:**
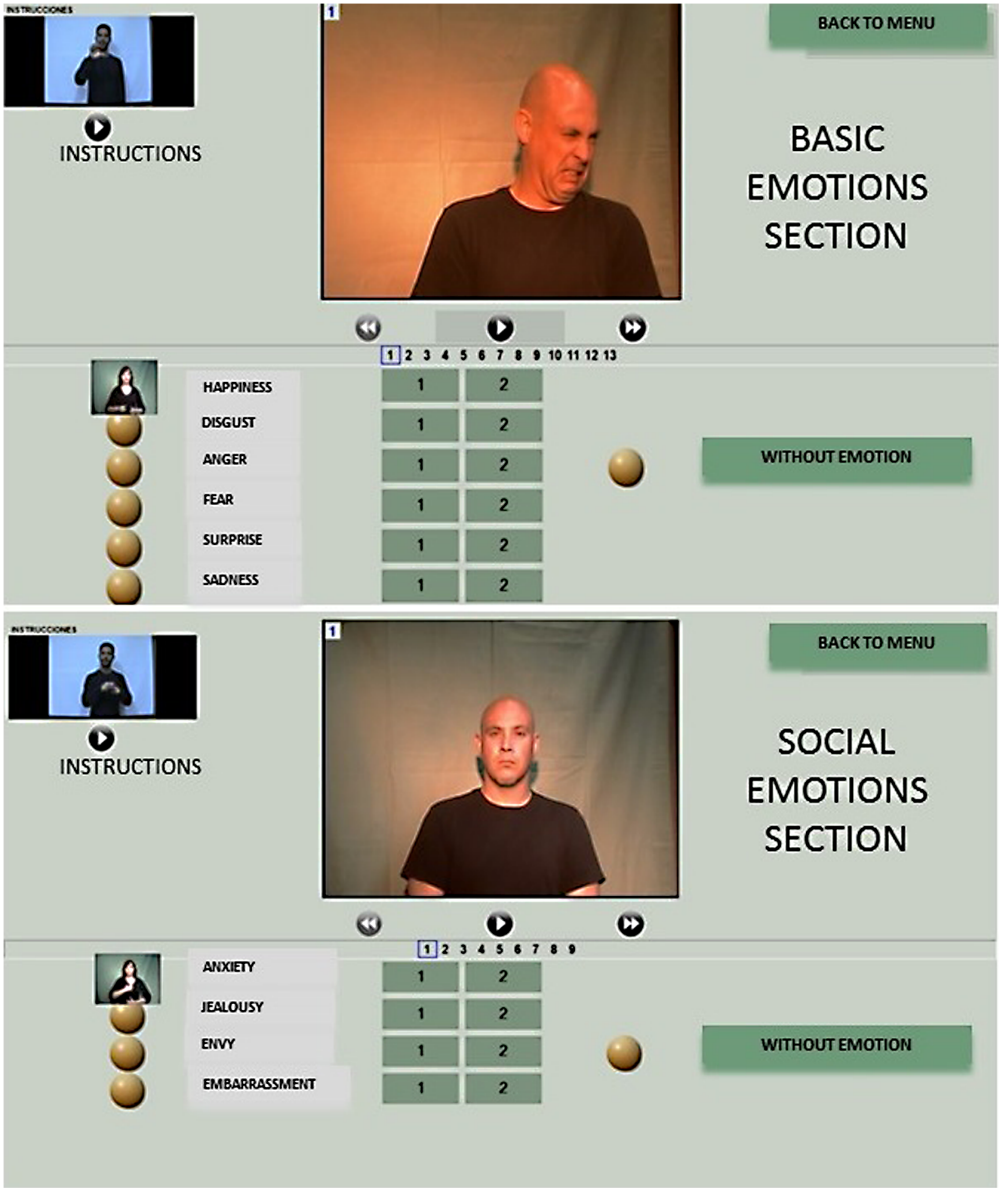
**A sample item for both basic and social emotional expression**. Both examples have been translated into English.

The final version has several inserted videos in the program interface. There are three types of videos (see **Figure 1**): (A) an instruction video at the top left of the interface with simultaneous oral and Spanish Sign Language (SSL) explaining how to use the program. In this video, the instructor asks participants to view all stimuli before starting to answer them, because there are two types of emotion stimuli (with different emotional expression intensity, high and low) for each basic emotion (fear, sad, surprise, anger, joyful, and disgust) and for each social emotion (anxiety, jealousy, envy, and embarrassment). In addition, there was one stimulus without emotional expression for each basic and social emotional test section. The right answer for each item was obtained via consensus among six deaf signal interpreters. The section on basic emotion contains 13 items and that of social emotion contains nine items; (B) a central bigger video with the stimuli to be answered below; and (C) a smaller video, which is presented when the yellow circle is clicked on. This video consists of an interpreter explaining what the displayed emotion is (using SSL, if the user wanted further information about the emotion label). If the participant’s answer matched the emotion and the intensity, then s/he obtains one point; if the answer matched the emotion but not the intensity, then it is scored as 0.5 point. The neutral item was scored 1.0 if the participant gave the correct answer—no emotion shown—and 0.0 for any other answer. Therefore, the possible score that could be obtained in the section on basic emotion ranges from 0 to 13 points, and the social emotion section from zero to nine points. Hence, the maximum total score is 22.0 points. Outcomes are presented in percentages to facilitate interpretation. Finally, the software generates an excel file with the answers given by participants.

The internal consistency obtained was highly moderate (Cronbach’s alpha 0.73) for the total scale, 0.69 for the basic emotions scale, 0.68 for the social emotions scale. However, Zumbo et al. (2007) recommended using polychoric correlations by omega coefficients as a better estimator of reliability for items with a categorical nature, where values can be interpreted similarly to the alpha coefficient. Following the authors’ suggestion, the calculated PERVALE-S omega values were 0.78 for the total scale, 0.75 for basic emotion, and 0.73 for social emotion. Intraclass correlation among expert referees (*n* = 6) for the software was 0.89 (*p* < 0.001), indicating a high level of agreement for each instrument item. Lastly, the correlation between sections and total PERVALE-S scores were calculated. The correlations between the basic and social emotion sections and the total scale were, respectively, *r* = 0.715 and *r* = 0.88 (both *p* < 0.001).

#### Fluid Intelligence (*Gf*)

Raven’s Standard Progressive Matrices test (RSPM; Raven et al., 1993) was administered to the participants. RSPM comprises 60 problems, and is divided into five sets (A–E) of increasing difficulty. Each set starts with easy problems and ends with more difficult ones. Each item contains a matrix of geometric design with one cell of the matrix removed, and there are six or eight alternatives given to insert in the place of the missing cell, one of which fits correctly. All participants were tested individually, without time limit, and RSPM instructions were given to DP using SSL by an interpreter. We used the achievement rate [(number of correct answers/60) × 100] as an index. Errors were not discounted, as in other intelligence tests. RSPM obtained good internal consistency in this study (Cronbach’s alpha = 0.872).

#### Personality

Because we sampled participants aged 12 years and older, we decided to administer a Spanish adaptation of the HSPQ (High School Personality Questionnaire; Cattell and Cattell, 1995). This questionnaire also contained videos providing instructions on how to answer the test and explanations of each of the 14 HSPQ scales. All deaf participants were instructed to watch the video before answering the HSPQ. HP received a brief verbal explanation about how to proceed. According to our experience with frequency adverbs, explained above, we used an answer scale with three levels (1: a little; 2: medium; and 3: much). This test assesses 14 personality factors, which are summarized in **Table 2**.

**Table 2:** High School Personality Questionnaire (HSPQ) Factors.

HSPQ factor	Low score = close to 1	Dimension	High score = close to 3
A	Reserved	Warmth	Outgoing
B	Less	Intelligence	More
C	Changeable	Emotional stability	Stable
D	Phlegmatic	Excitability	Excitable
E	Compliant	Dominance	Dominant
F	Sober	Cheerfulness	Enthusiastic
G	Rebellious	Consciousness	Rule-bound
H	Shy	Boldness	Extroverted
I	Realistic	Sensivity	Sensitive
J	Vigorous	Withdrawal	Doubtful
O	Placid	Apprehension	Apprehensive
Q_2_	Dependent	Self-sufficiency	resourceful
Q_3_	Undisciplined	Self-discipline	Controlled
Q_4_	Relaxed	Tension	Tense

*Scores on each item range from 1 to 3*.

#### Adaptation School Criteria for Deaf

We designed a questionnaire to administer to the educators at the Special Education Center for the Deaf, which all the DP in our study attended. All of them belonged to this school, or still belong to it. We asked four educators at the school to rate from 1: “nothing related to him/her” to 6: “completely related to him/her.” These professionals (teachers and counselors) had known each DP in our study for at least 2 years. We addressed five questions to be answered independently for each professional. These questions were related to: (1) adaptation to school rules; (2) impulsiveness control; (3) academic achievement; (4) peer acceptation; and (5) degree of conflict with others. Intraclass correlation for each question among the educators were 0.92, 0.91, 0.84, 0.87, 0.88, respectively. These indices indicated a high degree of agreement among raters.

### Procedure

All measures were presented in the following order: HSPQ, RSPM, and PERVALE-S. Signers and lip-reading DP below 17 years old performed all the tests in the Center for Deaf Education (Jerez, Spain). HP and DP 18 years old and above performed the tests at the Emotional Intelligence Lab of the University of Cadiz (Puerto Real, Spain). An interpreter was always present with the deaf sample, even if they did not require her help. All measures were performed individually. HP performed the same version of each measure, except with a prior verbal explanation. Participants received a brief report about their scores on *Gf* and emotional perception.

## Results

### Emotional Perception Achievement Among Groups

Our primary objective was to investigate whether DP perform worse than HP using an appropriate emotional perception tool developed for DP. **Table 3** shows the scores obtained in each section of the PERVALE-S by linguistic group.

**Table 3:** Means and *SD* of PERVALE-S scores by linguistic group.

Linguistic code	Total		Basic emotions		Social emotions	
	*M*	*SD*	*M*	*SD*	*M*	*SD*
Just signer deaf (*n* = 18)	79.13	12.56	84.19	13.12	74.07	16.39
Lip-reading deaf (*n* = 8)	70.30	11.03	83.65	6.74	56.94	21.36
Hearing (*n* = 30)	79.44	11.28	86.28	12.27	72.59	15.84

*Numbers represent percentages*.

**FIGURE 2:**
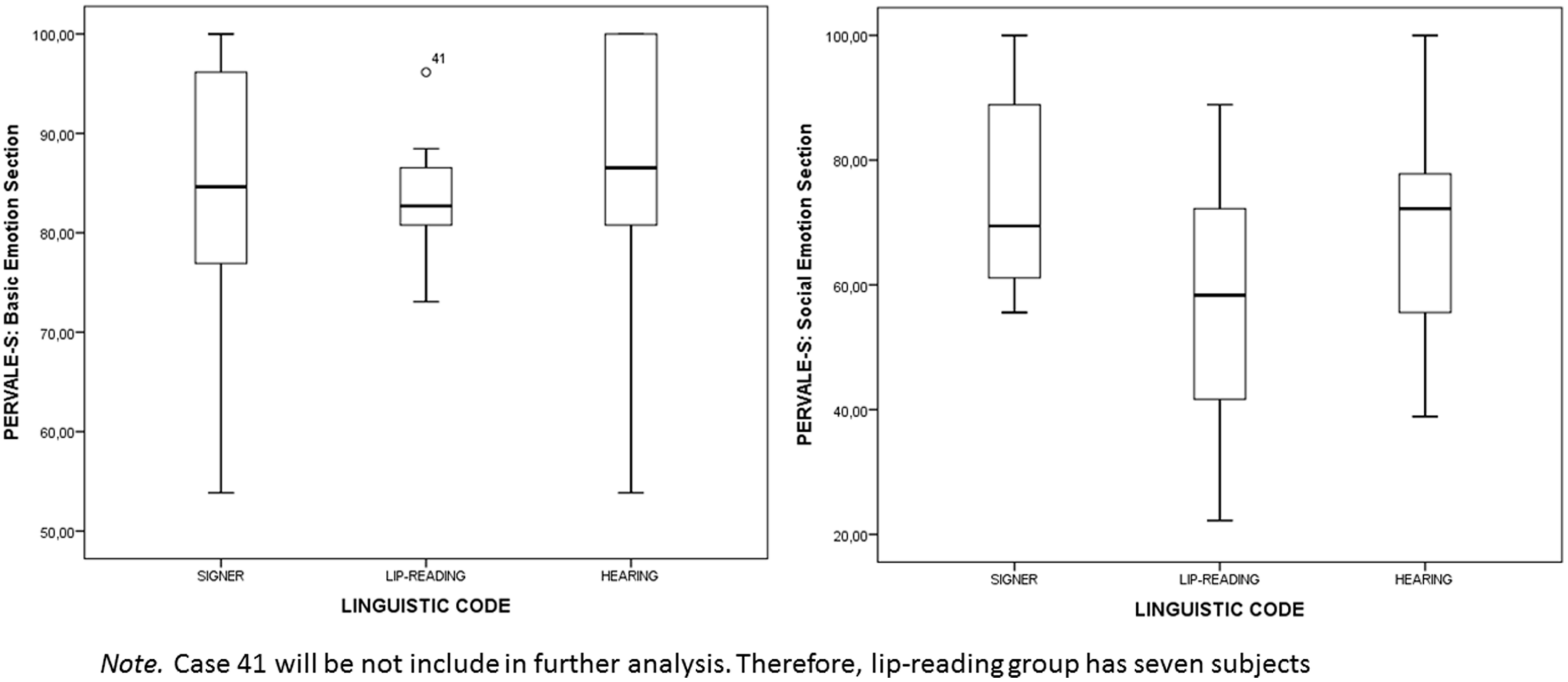
**Box-plot of bot PERVALE-S sections by linguistic code group**.

Lip-reading DP performed worse, especially in the social emotion section. **Figure 2** provides extra information about performance by PERVALE-S section and linguistic group.

However, the non-parametric equivalent of the ANOVA, the Kruskall–Wallis test, did not reveal any significant difference between the three groups. The findings were as follows: (a) total score χ^2^ = 3.81 (*p* = 0.15); (b) basic emotions section χ^2^ = 1.09 (*p* = 0.58); and (c) social emotions section χ^2^ = 3.56 (*p* = 0.17). We were also interested in displaying both basic and social confusion matrices by linguistic group. According to the results of the Kruskall–Wallis test, we illustrate both matrices using the entire sample, rather than by linguistic group (see **Tables 4** and **5**).

**Table 4:** Confusion matrix for basic emotions (frequency response).

ITEM	1	2	3	4	5	6	7	8	9	10	11	12	13	Success	Success	Error
1	**50**	5											1	89.3	8.9	1.8
2	7	**44**	1						1	3				78.6	12.5	9.0
3			**40**	14	2									71.4	25.0	3.6
4			1	**55**										98.2	1.8	0
5					**42**	13							1	75.0	23.2	1.8
6				1	9	**45**	1							80.4	16.1	3.6
7							**32**	4	15	2	2		1	57.1	7.1	35.8
8							7	**49**						87.5	12.5	0
9									**45**	11				80.4	19.6	0
10		6				1			6	**43**				76.8	10.7	12.5
11					2	1	1		1		**41**	9	1	73.2	16.1	10.8
12			5	2		1	1	2			9	**36**		64.3	16.1	19.6
13	3										2		**51**	91.1	-	8.9

*Success percentage rate, medium success percentage rate (matching the emotion but not the intensity; ½ success), and error percentage rate. The correct frequencies are displayed in boldface*.

*Emotion 1 is less intensely expressed than Emotion 2*.

*1: Happiness 1; 2: Happiness 2; 3: Disgust 1; 4: Disgust 2; 5: Angry 1; 6: Angry 2; 7: Fear 1; 8: Fear 2; 9: Surprise 1; 10: Surprise 2; 11: Sadness 1; 12: Sadness 2; 13: Neutral or no emotion expressed*.

**Table 5:** Confusion matrix for social emotions (frequency response).

Item	1	2	3	4	5	6	7	8	9	Success	Success	Error
1	**48**	6			1		1			85.7	10.7	3.6
2	1	**50**	1		1	1		2		89.3	1.8	9.0
3	2	1	**24**	8	14	6			1	42.9	14.3	42.9
4		1	12	**18**	7	18				32.1	21.4	46.4
5	1		16	4	**27**	5	2		1	48.2	8.9	42.9
6	2	2	5	18	13	**15**	1			26.8	23.2	50.0
7	2						**47**	7		83.9	12.5	3.6
8							10	**46**		82.1	17.9	0
9		1	3		2				**50**	89.3	-	10.7

*Success percentage rate, medium success percentage rate (matching the emotion but not the intensity; ½ success), and error percentage rate. The correct frequencies are displayed in boldface*.

*Social Emotion 1 is expressed less intensely than Social Emotion 2*.

*1: Anxiety 1; 2: Anxiety 2; 3: Jealousy 1; 4: Jealousy 2; 5: Envy 1; 6: Envy 2; 7: Embarrassment 1; 8: Embarrassment 2; 9: Neutral or no emotion expressed*.

In order to identify the influences of age and *Gf*, we also analyzed their relationship with PERVALE-S scores. For signers (*n* = 18; mean age = 21.06, *SD* = 6.31), the age by task section correlations were *r*_age-basic_ = 0.25 (*p* > 0.05), *r*_age-social_ = 0.48 (*p* < 0.01); and the age with *Gf* (*M* = 76.11, *SD* = 20.46) correlation was *r* = 0.69 (*p* < 0.001). For lip-readers (*n* = 8; mean age = 16.86, *SD* = 5.73), the age by task section correlations were *r*_age-basic_ = -0.09 (*p* > 0.05), *r*_age-social_ = 0.61 (*p* < 0.001), and the age-*Gf* correlation was *r* = 0.08 (*p* > 0.05). Finally, for HP (*n* = 30; mean age = 23.4, *SD* = 4.48), the age by task section correlations were *r*_age-basic_ = 0.01 (*p* > 0.05), and *r*_age-social_ = 0.25 (*p* > 0.05).

Regarding main mistakes by language code, we found different percentages in the errors made within the signer group when identifying emotions: joy, fear, surprise, and “no emotion” (22.2% of the signer sample), jealousy and envy (38.9% of sample). Different percentages emerged among lip-readers: joy and surprise (42.9%), fear (57.1%), anxiety (28.6%), envy, jealousy, and embarrassment (57.1%). The percentages among HP were 40% for fear, 16.7% for surprise and sadness, and 46.7% for jealousy and envy. *Post hoc* Kruskall–Wallis analysis confirmed significant differences between groups in according next items: joy-1 (*p* = 0.015), anger-2 (*p* = 0.02), and no emotion in the basic emotion section (*p* = 0.03).

**Table 6:** Correlations between PERVALE-S and age, sex, fluid intelligence (*Gf*), and HSPQ measures (*N* = 55, after eliminating case 41).

	*M*	*SD*	Total	Basic	Social
Age	21.68	5.65	**0.391^∗∗^**	0.124	**0.447^∗∗^**
Sex (1: male; 2: female)	-	-	0.101	0.132	0.048
Raven’s Standard Progressive Matrices test (RSPM) *Gf*	80.71	15.12	**0.288^∗^**	0.163	**0.281^∗^**
HSPQ A: Warmth	2.27	0.73	0.231	0.233	0.157
HSPQ B: Intelligence	2.43	0.60	0.142	0.229	0.039
HSPQ C: Emotional stability	2.38	0.75	0.087	0.036	0.094
HSPQ D: Excitability	2.14	0.70	-0.066	-0.121	-0.008
HSPQ E: Dominance	1.91	0.61	-0.252	-0.099	-**0.276^∗^**
HSPQ F: Cheerfulness	2.14	0.70	0.141	0.040	0.165
HSPQ G: Consciousness	2.43	0.68	0.253	**0.347^∗∗^**	0.110
HSPQ H: Boldness	1.96	0.66	-0.057	-0.096	-0.013
HSPQ I: Sensivity	2.32	0.57	0.131	0.094	0.115
HSPQ J: Withdrawal	1.64	0.70	-0.051	-0.066	-0.025
HSPQ O: Apprehension	1.68	0.66	-0.219	-0.242	-0.135
HSPQ Q2: Self-sufficiency	1.38	0.52	-**0.265^∗^**	-0.196	-0.228
HSPQ Q3: Self-discipline	2.21	0.59	0.089	0.180	0.000
HSPQ Q4: Tension	1.71	0.73	-0.237	-**0.303^∗^**	-0.118

*Significant rho relationships are in boldface*.

*Correlations were calculated using Spearman’s rho. ^∗^p < 0.05, ^∗∗^p < 0.01; one tailed*.

Next, we checked whether both PERVALE-S fits into the EK construct and EPA is related to some personality traits. **Table 6** shows the correlations between PERVALE-S sections and age, sex, *Gf*, and personality traits.

Our last objective was to test the expected relationship between PERVALE-S sections and adaptation school criteria. **Table 7** shows the rho correlations.

The results show that personality and *Gf* do not belong to the EK construct. However, *Gf* was not related to any adaptation criteria. Among personality traits, we found almost significant relationships: adaptation to school rules and impulsiveness control were negatively related to tension (Q_4_) *r*_tension-adaptation_ = -0.50, *r*_tension-impulsiviness_ = 0.62, *p* < 0.05. Academic achievement was positively related to sensitivity (I) *r* = 0.54, *p* < 0.01; and negatively related to apprehension *r* = -0.48, self-sufficiency *r* = -0.50, and tension (Q_4_) *r* = -0.43 (last three *p* < 0.05). Peer acceptation was positively related to sensitivity (I) *r* = 0.42, *p* < 0.05. Unrest was positively related to dominance (E) *r* = 0.44 and tension (Q4) *r* = 0.51 (both *p* < 0.05).

However, Mann–Whitney analysis did not show any significant difference between the genders for all ratings.

## Discussion

This pilot sudy; only signer DPs are increasingly uncommon now due to the advent of cochlear implantstudy had some difficulties recruiting lip-readers, because most of them did not belong to the deaf school anymore. In addition, we could recruit only 18 signers for the pilot st and the inclusion of verbal language education in the school curriculum. However, we decided not to include DPs with cochlear implants because most of them were below 12 years old. Finally, we included a sample of HP to compare their performance with the deaf sample.

### Testing PERVALE-S among Linguistic Groups

The main goal of the pilot study was to test the new version of PERVALE-S. Generally, participants performed better in the basic emotion section compared to the social one. Emotional development studies have pointed out that social emotion development takes more cognitive effort and time than basic emotions (Arsenio, 2003). Indeed, EPA—the first branch of the EI ability model (Mayer and Salovey, 2007)—forms the basis on which other branches (using, understanding, and managing) grow. The ability to perceive basic emotions (matching the correct emotion) takes about 6–8 years to develop, while social emotions require about 12 years (Zeidner et al., 2003; Mestre et al., 2007). This corroborates with our finding that age is correlated to the social emotion section but not the basic emotion section (*r* = 0.447, *p* < 0.01).

Regarding the first objective of this pilot study, the correct answer rate by section was around 70%, which is an appropriate score for future standards. Except among lip-readers in the social emotion section, who were also younger than the other participants. Even so, the non-parametric ANOVA did not reveal any difference between linguistic groups. However, the analysis of the influence of age by group revealed some relationships, especially among signers. In this case, age was related to both sections. In the lip-reading group, age was related to just the social emotion section. Probably, both age and sample size compromised this finding in the lip-readers group. No age by section relationships were found in the hearing group. Cautiously, our results are comparable to those of previous studies (Dyck and Denver, 2003; González et al., 2011). Thus, DP probably need more time to identify the emotion expressed compared to HP, perhaps due to fewer opportunities to obtain experience in matching emotional expressions (Alegria and Lechat, 2005). Another complementary explanation comes from the lack of abilities for listening. DP need to compensate for their disabilities in hearing to enhance their accuracy in emotional perceptions. For this purpose, they need to spend more time to obtain similar performance standards (Letourneau and Mitchell, 2011). *Gf* could be a mediating variable in this relationship, as catalyst for emotional learning (Farrelly and Austin, 2007; Belke et al., 2008; Ono et al., 2011).

**Table 7:** Correlations between PERVALE-S and adaptation criteria.

Criteria	*M*	*SD*	Basic emotion	Social emotion
Adaptation to school rules	3.78	1.15	0.17	0.21
Impulsiveness control	3.45	1.27	**0.41^∗^**	-0.08
Academic achievement	3.26	1.23	0.11	0.16
Peer acceptance	3.33	1.01	0.01	0.06
Unrest	2.15	1.19	-**0.45^∗^**	-**0.39^∗^**

*Significant relationships are in boldface (DP: *n* = 26)*.

*Correlations were calculated using Spearman’s rho. ^∗^p < 0.05 one tailed*.

There is an interesting theoretical debate about facial expression of emotion and the meaning of motor expression (Nelson and Russell, 2013). However, it is also necessary to identify the underlying determinants and production mechanisms in order to widely encompass the nature of this process, and choose fundamental assumptions and predictions regarding the patterning of facial expressions for different emotions (Scherer et al., 2013). The PERVALE-S items were based on idiosyncratic emotional expressions for the deaf from the south of Spain. For this purpose, we worked with six expert deaf interpreters and confirmed that they highly agreed on the right answer for each item (intraclass correlation was 0.89, *p* < 0.001). We also report the advice given by interpreters about this idiosyncratic emotional culture in DP (Lederberg et al., 2013); thus, the PERVALE-S items might not be appropriate for other cultures, however, HP performed similarly to DP.

The confusion matrices showed that many mistakes were made in the social emotion section. In the basic emotion section, the deaf participants had difficulty identifying joyfulness (42.9% for lip-readers and 22.2% for signers) which is unusual. In fact, it is the easiest emotion to identify for children (Mestre et al., 2011). However, it is normal to have difficulty identifying surprise and fear (57.1% for identifying fear and 42.9% for surprise among lip-readers; Ekman, 1999; Jessen et al., 2012). HP erred mainly on fear when it was expressed with low intensity (40%). There were similar mistakes from the three groups in the social emotion section. Jealousy and envy were less likely to be identified correctly (from 36.7 to 57.1%, respectively). This outcome is interesting because it bolsters an idea pointed out by Salovey and Rodin (1988). The authors reported that jealousy seemed more intense than envy; moreover, both emotions seemed to be experienced practically in the same way. Hence, it is more a question of quantitative than qualitative experience (for more information, see Salovey, 1991). In other words, jealousy and envy require considerable cognitive development to be perceived correctly (Oatley and Johnson-Laird, 2014). Indeed, the relationship found between *Gf* and social emotion in this study (*r* = 0.28, *p* < 0.05) hints at a mediating role of intelligence in the perception of social or complex emotion in DP.

### Does PERVALE-S Fit into the EK Construct?

Emotional knowledge is a classic topic for emotional perception in children; however, it can also be used for other ages, for instance, sections A and E of MSCEIT (Mayer et al., 2003; for cross-cultural validation see Karim and Weisz, 2010). Critics have recently discussed if it is possible to measure the EI framework entirely (Maul, 2012a,b). PERVALE-S is a special instrument for measuring the first branch (perceiving).

Gender has been reported as an important variable in EI (Castro-Schilo and Kee, 2010), and in emotional perception (Chiaburu and Gray, 2008). In this study, gender was not significant because of the sample age. Future studies should confirm our results of no differences between groups.

Another question is how other EK measures have been computed. Take, for instance, the EMT (Emotional Match Task, Morgan et al., 2009). This instrument has been validated in Spain (see Alonso-Alberca et al., 2012) with a good reliability and promising validation processes. Nevertheless, some aspects of this task are still under debate. Some EMT items are computed the same way across different population subsets. For example, in the first part of EMT there are two ambiguous items, item number 8 and item number 11, the answers “sad” or “anger” have the same valor (they both are computed as “1”). Both emotions had similar accuracy rates although sadness and anger have different facial expressions (Ekman, 1999). In our opinion, this decision favors the psychometric properties of the instrument. In contrast, we believe that an emotional perception instrument should encompass different levels of achievement, as in an exam (Seal et al., 2009). Verbal intelligence might be used as a screening factor in future studies on emotion perception in DP, while *Gf* (e.g., Raven’s measure) should be tested as a mediating variable in EK score, even among HP. It is too early to recommend PERVALE-S as a tool for assessing emotional perception in DP. As cochlear implants are increasingly prevalent, the next step would be to test the instrument on a sample with cochlear implants. Therefore, prosody is another factor to include in the study of emotional perception or EK.

Age and intelligence were related to PERVALE-S, but social emotion should be redesigned, removing at least the envy items following Salovey and Rodin’s (1988) suggestions (quantitative difference). Another topic under debate is whether newborn deaf should be implanted, according to pilot study findings, these results should not be taken as support for the defenders of cochlear implant devices for deaf. However, we assume that technology will improve tremendously in the future, and that “metallic sounds” will be controlled. If DP indeed decode emotions similarly to HP, then another social adaptation barrier will disappear.

### The Role of Personality Traits

In fact, our personality test adapted for the deaf might be considered as semantic differential with three levels of agreement (1: “a little”, 2: “medium”, 3: “much”). We used HSPQ traits and created a pptx file embedded with videos. All participants understood how to fill it. This was another challenge because we did not assume that the deaf participants would understand the 144-item HSPQ.

We founded that participants who scored lower in the social emotion section perceived themselves as more dominant (*r* = -0.276, *p* < 0.05). Dominance and EPA are included in the hypothesis of subordination (Snodgrass, 1992), which states that the increased capacity of women (and compliant people) to perceive emotions is due to traditional social subordination which they have been or are being subjected to (for further reading see Elfenbein et al., 2002). The key idea of this hypothesis is that it is more important for subordinates to understand the emotions of those whom they are subordinated to (Keltner et al., 2003).

Regarding the basic emotion section, this subscale was positively related to consciousness (*r* = 0.347, *p* > 0.01) and negatively related to tension (*r* = -0.265, *p* < 0.05). Consciousness (and unconsciousness emotion processes) is attracting interest from emotional perception researchers (see Barret et al., 2005). People who pay attention to their tasks (as PERVALE-S) tend to score higher in this performance test as well (Matsumoto et al., 2000; Barret et al., 2005; Matsumoto, 2006). In contrast, neuroticism or tension predicts lower performance in emotional perception tasks (Matsumoto et al., 2000). Finally, the HSPQ trait of self-sufficiency was negatively related to overall PERVALE-S performance (*r* = -0.265, *p* < 0.05). Participants who prefers to work in groups, instead of alone, obtained better scores in the instrument. EI abilities are related to social interactions (Lopes et al., 2004, 2011; Grant et al., 2014). This is consistent with the relationship found in the present study. This may be because individuals who perceive themselves as easy-going are more interested in developing their social competence (Gross, 1998).

### Adaptation Criteria

In order to determine the predictive validity of the instrument, we asked four teachers who were familiar with the deaf participants to rate them along five social adaptation criteria. This strategy was used successfully to determine the predictive validity of EI (Mestre et al., 2006; Lopes et al., 2011, 2012). The level of agreement for each criterion was appropriate. Hearing participants were excluded from posterior multiple regression analysis. The final sample for this part of the study was 26 deaf participants. Stepwise multiple regression analysis revealed that both basic emotion section scores and age were related to impulsiveness control and to unrest. Traditionally, impulsiveness has been related to being able to regulate one’s emotions in school (Gross and Thompson, 2007). The consulted teachers agreed to consider this point of view. The deaf participants who were rated as being able to control their impulse better also scored higher in the basic emotion section. Unrest or conflict was also related negatively to basic emotions and age after regression analysis. This relation between emotional competence and unrest has been described previously, especially among males (Mestre et al., 2006; Lopes et al., 2012). Thus, the finding of this relationship in our study might be due to more than half the sample being male (68%). However, the Mann–Whitney analysis did not reveal this gender influence.

Despite these findings, we recommend prudence in the interpretation of the current results. PERVALE-S is still under review and predictive validity investigations are necessary. Currently, the instrument is being used to train emotional competence among DP.

## Conclusion

### Limitations and Strengths

The sample size and the lack of similar studies using an emotional perception instrument adapted for DP is a strong limitation. The research design did not allow the making of causal inferences, despite the multiple regression analysis used. Nevertheless, this was a pilot study whose main objective was to test the new instrument and derive standard scores for future investigations with DP. To develop an instrument for the deaf was a challenge, as well as adapting the HSPQ.

Removing verbal intelligence influences from the pilot study and developing an emotional perception instrument for DP minimized traditional differences in the emotional perception assessment between DP and HP. This pilot study provides an interesting contribution to the literature on emotional perception and deaf. In addition, it is a first step to assess EI in DP. The main problem in assessing EI is verbal intelligence (especially in the third branch: understanding emotions, see Beck et al., 2012), and verbal intelligence is a measure of crystallized intelligence (Mackintosh, 2003). Because of these influences, some emotionally competent people obtained low scores in some EI performance test as MSCEIT (Amitay and Mongrain, 2007; Nafukho, 2010). Therefore, the development of instruments such as PERVALE-S allows the separation of the influence of verbal ability on non-linguistic EI studies.

Emotional knowledge and many emotional perception tests have not included social emotion stimuli. This might be due to problems with using static stimuli to simulate existential emotional states, such as anxiety, jealousy, and embarrassment (Krumhuber et al., 2013). Some social emotions are existential, for instance anxiety, jealousy, embarrassment, or envy (Lazarus and Lazarus, 1996). Indeed, participants often committed high error percentages (Lazarus and Lazarus, 1996). Avoiding social emotion stimuli does not encompass the whole emotional sphere and how to face it (Summerfeldt et al., 2006). Moreover, the relationship between *Gf* and social emotion should be explored with a bigger sample. Previous studies have suggested the role of non-linguistic abilities in the EI (see Albanese et al., 2010). Another strength is that PERVALE-S fits in the current perception theories (top–down versus bottom–up).

### Generalizability and Heuristics

It was difficult to recruit the deaf sample, due to the changing situation regarding DP. For example, Spanish deaf students are receiving an inclusive education instead of in a special school (Kelman and Branco, 2009). Furthermore, there is ongoing debate about whether cochlear implants should be given to all newborn deaf (Kermit, 2009). Some Spanish deaf communities are awaiting studies that support their point of view against cochlear implantation (Herrero et al., 2009), but this pilot study is not an answer for or against of the cochlear implant. Our point of view is that the deaf community claims for a major interest on their issues from HP rather than vice-versa. Moreover, DP should not perceive the cochlear implant as a menace for their own culture or way of life (Wang et al., 2011). However, this research itself might help to change some HP stereotypes about the affectivity of DP, due to their emotional performances were similar to HP.

Finally, we considered cognitive information processing in DP (just iconic inputs) when the new version of PERVALE-s was developed (i.e., reducing the levels of the answer scale from 5 to 3); however, we are unsure if this software could be generalized to another deaf community due to the special dialect for Southern SSL. However, hearing participants seemed to understand most of the basic emotions and committed the same errors as the deaf in the social emotion section (envy and jealousy). This software is easily translatable for any researcher interested in the software. In addition, the videos could be replaced.

## Conflict of Interest Statement

The authors declare that the research was conducted in the absence of any commercial or financial relationships that could be construed as a potential conflict of interest.

## Footnotes

1 According to wikipedia, “a confusion matrix, also known as a contingency table or an error matrix, is a specific table layout that allows visualization of the performance of an algorithm, typically a supervised learning one (in unsupervised learning it is usually called a matching matrix). Each column of the matrix represents the instances in a predicted class, while each row represents the instances in an actual class. The name stems from the fact that it makes it easy to see if the system is confusing two classes (i.e., commonly mislabeling one as another).”
